# Age-dependent development of liver fibrosis in *Glmp*^*gt/gt*^ mice

**DOI:** 10.1186/s13069-016-0042-4

**Published:** 2016-04-28

**Authors:** Cecilie K. Nesset, Xiang Yi Kong, Markus Damme, Camilla Schjalm, Norbert Roos, Else Marit Løberg, Winnie Eskild

**Affiliations:** Department of Bioscience, University of Oslo, Oslo, Norway; Research Institute for Internal Medicine, University of Oslo, Oslo, Norway; Institute of Clinical Medicine, Faculty of Medicine, University of Oslo, Oslo, Norway; K.G. Jebsen Inflammation Research Centre, University of Oslo, Oslo, Norway; Institute of Biochemistry, Christian-Albrechts-Universität Kiel, Kiel, Germany; Department of Immunology, University of Oslo, Oslo, Norway; Department of Pathology, Oslo University Hospital Ullevaal, Oslo, Norway

**Keywords:** Liver fibrosis, Transgenic mouse model, Liver tumors, Oval cells, Inflammation

## Abstract

**Background:**

Mice lacking glycosylated lysosomal membrane protein (*Glmp*^*gt/gt*^ mice) have liver fibrosis as the predominant phenotype due to chronic liver injury. The *Glmp*^*gt/gt*^ mice grow and reproduce at the same rate as their wild-type siblings. Life expectancy is around 18 months.

**Methods:**

Wild-type and *Glmp*^*gt/gt*^ mice were studied between 1 week and 18 months of age. Livers were analyzed using histological, immunohistochemical, biochemical, and qPCR analyses.

**Results:**

It was shown that *Glmp*^*gt/gt*^ mice were not born with liver injury; however, it appeared shortly after birth as indicated by excess collagen expression, deposition of fibrous collagen in the periportal areas, and increased levels of hydroxyproline in *Glmp*^*gt/gt*^ liver. Liver functional tests indicated a chronic, mild liver injury. Markers of inflammation, fibrosis, apoptosis, and modulation of extracellular matrix increased from an early age, peaking around 4 months of age and followed by attenuation of these signals. To compensate for loss of hepatocytes, the oval cell compartment was activated, with the highest activity of the oval cells detected at 3 months of age, suggesting insufficient hepatocyte proliferation in *Glmp*^*gt/gt*^ mice around this age. Although constant proliferation of hepatocytes and oval cells maintained adequate hepatic function in *Glmp*^*gt/gt*^ mice, it also resulted in a higher frequency of liver tumors in older animals.

**Conclusions:**

The *Glmp*^*gt/gt*^ mouse is proposed as a model for slowly progressing liver fibrosis and possibly as a model for a yet undescribed human lysosomal disorder.

**Electronic supplementary material:**

The online version of this article (doi:10.1186/s13069-016-0042-4) contains supplementary material, which is available to authorized users.

## Background

Mammalian liver plays a vital role in the maintenance of body homeostasis. This homeostatic activity comprises the supply of energy substances and a number of blood components for which the liver is the only or major site of synthesis, i.e., serum albumin [1]. In addition, the liver removes a wide selection of potentially harmful substances arriving from the gut via the portal vein as well as substances, which are needed for synthetic purposes. Clearance from the circulatory system of various macromolecules and particles occurs by endocytosis, generally leading to degradation of endocytosed substances in the lysosomes followed by excretion or recycling of the degradation products in synthetic pathways. The liver is one of the most active metabolic organs of the body, having a high turnover of its constituents. Degradation of intracellular substances proceeds via the autophagic pathway for which the lysosome is also the endpoint [2].

Proper functioning of the lysosomes is essential to the homeostatic activity of hepatocytes and often also to their survival [3]. A heterogeneous group of more than 50 congenital metabolic disorders characterized by the lack or dysfunction of a protein within the endo-lysosomal pathway has been described and categorized as lysosomal disorders [4–11]. Most of these proteins are degradative enzymes or membrane proteins [10, 11]. These lysosomal disorders have widely varying phenotypes; however, symptoms are often observed in tissues with low proliferation capacity, such as the brain, or in cell types with a high substrate turnover such as hepatocytes [7, 8, 12, 13]. A consequence of lysosomal disorder is gradual accumulation of undegraded or unrecycled substances leading to increasing pathogenic effects at the cellular and organ level eventually leading to systemic dysfunction [14, 15]. Depending on the type and degree of such a lysosomal disorder, patients are faced with a shorter life expectancy [7, 8].

A frequent clinical manifestation in many lysosomal disorders is liver fibrosis [9], a consistent result of almost every type of prolonged or chronic liver injury [16]. Fibrogenesis is characterized by hepatocyte apoptosis/necrosis and activation of Kupffer cells and hepatic stellate cells [16–19]. These events lead to release of a wide array of cytokines (e.g., TGF-β, TNF-α, and PDGF), leading to infiltration of leukocytes and further activation of stellate cells [17, 20, 21]. A state of inflammation results, often accompanied with oxidative stress [17, 20, 21].

Fibrosis is a wound healing process during which the balance between the extracellular matrix components is shifted partly due to changes in expression of matrix genes, such as collagen I, and partly due to changes in expression of an array of matrix degrading enzymes and their inhibitors, leading to accumulation of stiff, highly cross-linked filamentous collagen structures [20, 22, 23]. If untreated, liver fibrosis may proceed to liver cirrhosis, cancer, liver failure, and eventually death [24]. Worldwide chronic liver injury is one of the major causes of morbidity and mortality [25].

The progression from chronic liver injury to liver fibrosis and eventually cirrhosis depends on the etiology [17, 19]. Having access to animal models representing these different pathologies is important in order to lay the foundation for appropriate treatment strategies. Mouse models representing spontaneous liver fibrosis starting early in life but with a slow progression are scarce [26, 27]. We recently reported the successful generation of a mouse model lacking expression of the glycosylated lysosomal membrane protein (GLMP) [28]. The physiological function of this protein is unclarified, yet it appears to be essential to a healthy liver. By the age of 6 months, the *Glmp*^*gt/gt*^ mouse had developed a marked liver fibrosis associated with hepatic cell death, oxidative stress, active fibrogenesis, and activation of liver progenitor cells [28].

The present study was undertaken to explore the age-dependent development of liver fibrosis in *Glmp*^*gt/gt*^ mice. This report shows that development of liver fibrosis is initiated shortly after birth in *Glmp*^*gt/gt*^ mice and progresses to a peak around 4 months of age. These mice are long-lived but develop liver tumors at later stages in life. Expression of inflammation markers and genes responsible for alteration of the extracellular matrix increases from 2 weeks of age. Activation of hepatic stellate cells and hepatic progenitor cells is especially prominent around 3 to 4 months of age. Further, expression of genes for inhibitors of cell cycle progression is increased. At the age of 18 months, a majority of the *Glmp*^*gt/gt*^ mice had developed liver tumors. We propose that the *Glmp*^*gt/gt*^ mouse model may prove to be very useful for studies of slowly progressing liver fibrosis and possibly as a model for a yet undescribed lysosomal disorder.

## Methods

### Animals and handling

All animal experiments were reviewed and approved by the Norwegian Animal Research Authority and performed according to national laws and regulations. Generation of wild-type (WT) and *Glmp*^*gt/gt*^ mice has been described [28]. The animals were housed in an approved animal facility with access to standard rodent chow and water ad libitum unless otherwise stated. Biological samples from both males and females were collected at designated age-points (newborn—18 months). Samples not immediately processed or used for analyses were snap-frozen in liquid N_2_ and stored at −80 °C until use.

### Serum and hematological analysis

Blood was collected by cardiac puncture, coagulated at room temperature before centrifugation at 1500*g* for 20 min. Serum was collected and immediately analyzed for serum alanine transaminase [29], aspartate transaminase (AST), γ-glutamyl-transferase, bile acid, bilirubin, and albumin concentrations at The Central Laboratory, Department of Basic Sciences and Aquatic Medicine, Norwegian School of Veterinary Science. Serum levels of TGF-β were assessed using enzyme-linked immunosorbent assay (#MB100B, R&D systems, Minneapolis, MN, USA) according to manufacturer’s procedure.

Blood was collected from 4.5-month-old male WT and *Glmp*^*gt/gt*^ mice by cardiac puncture into micro-vessel EDTA tubes (Terumo Europe, Leuven, Belgium). Hematological analyses were performed by The Central Laboratory, Department of Basic Sciences and Aquatic Medicine, Norwegian School of Veterinary Science.

### Histochemistry

Mouse tissues were fixed in 10 % neutral buffered formalin overnight, processed into paraffin blocks, and sectioned into 4-μm-thick slices. Liver sections were stained with either hematoxilin and eosin (H&E) or acid fuchsin orange G (AFOG) according to standard techniques.

### Immunohistochemistry

Formalin-fixed paraffin-embedded sections were deparaffinized in xylene, rehydrated in a graded series of ethanol, and demasked in a microwave oven for 24 min in Tris/EDTA (TE) buffer (pH = 9.1). Antibodies for immunohistochemistry were Rabbit monoclonal anti-Ki67 (1:500, SP6, GTX16667, Cytotech ApS, Hellebæk, Denmark). Sections were counterstained with hematoxylin, and the antigen-antibody reaction was visualized with Dako EnVision horse radish peroxidase system using 3,3′-diaminobenzidine as the chromogen (K4007, DAKO North America Inc., Camarillo, CA, USA).

Livers from four individual animals of each genotype and age group were used for quantification of proliferating hepatocytes. From each animal, two representative liver tissue blocks were used for obtaining four randomly collected slices including at least 1000 hepatocytes from each liver (imaged with ×40 obj.). The total number of proliferative hepatocytes, given as the sum of all positively Ki67 labeled hepatocyte nuclei, and the semi-quantitative estimation of the average number of hepatocyte nuclei included in a magnification field were determined by counting.

### Immunofluorescence

Immunofluorescence was performed as described previously [28]. Antibodies used for immunofluorescence were Rat monoclonal anti-A6 (cell culture supernatant 1:25) kindly provided by Dr. Valentina Factor [30] as primary antibody and Alexa-Fluor-633 as secondary antibody (Molecular Probes, Eugene, OR, USA), and sections were counterstained with DAPI. Estimation of immunofluorescence intensity was graded in a semi-quantitative summary.

### Transmission electron microscopy

Mice (*n* = 3, age 6 months) were subjected to perfusion fixation using HEPES buffer (0.1 M, pH 7.2–7.4) with 4 % formaldehyde and 2.5 % glutaraldehyde. Tissues were collected, cut into blocks of 1 mm^3^, transferred to new fixative solution, and kept at 4 °C overnight. The samples were rinsed 2 × 10 min in 0.1 M sodium cacodylate buffer prior to post-fixation (2 % OsO4 in 0.1 M sodium cacodylate buffer) for 1 h, and rinsed 5 × 10 min in distilled water before bulk staining with 1.5 % uranyl acetate (CH_3_COO)2UO_2_·2H_2_O) [29] in distilled water for 30 min in the dark. Fixed tissue samples were dehydrated in a graded series of ethanol for 10 min each (70, 80, 90, 96 %), 4 × 15 min in 100 % ethanol and finally 2 × 10 min in propylene oxide. Following dehydration, tissue samples were infiltrated with epoxy:propylene oxide (1:1) for 30–60 min on a rotary shaker, evaporated overnight, and the following day incubated 1 h in pure epoxy before embedding in plastic capsules and polymerization at 60 °C. Five tissue blocks were selected per individual mouse. Ultrathin sections were obtained using a Leica Ultracut S microtome (Leica, Wetzlar, Germany) with diamond knife and collected on copper-coated grids before staining with lead citrate for 20 s. All electron micrographs were obtained with a CM100 transmission microscope (Philips, Amsterdam, The Netherlands) at 80 kV.

### Determination of Hydroxyproline

Briefly, frozen livers from WT and *Glmp*^*gt/gt*^ mice were ground into powder, hydrolyzed in 6 M hydrochloric acid (HCl) at 100 °C o/n followed by centrifugation at 13,000*g* for 10 min. Supernatants were diluted 1:2 with 4 M HCl. Liver hydroxyproline contents were determined using Hydroxyproline Assay kit (#QZBhypro2, QuickZyme Biosciences, Leiden, The Netherlands), according to manufacturer’s protocol.

### Gene expression analyses

RNA extractions from mouse liver were carried out according to the manufacturer using RNeasy Plus kit (Qiagen, Hilden, Germany). The expression of selected messenger RNA (mRNA) transcripts (Additional file 1: Table S1) was analyzed by quantitave PCR (qPCR) using a LightCycler 480 (Roche Diagnostics, Manheim, Germany) and LightCycler® 480 SYBR Green I Master Mix (Roche Applied Science). PCR efficiencies were experimentally determined for each primer pair. Relative gene expression was calculated using the ΔΔCt-method, with beta-actin and eukaryotic translation elongation factor 2 as reference genes.

### Statistical analysis

All results are expressed as mean ± s.e.m. All data were analyzed using two-tailed *t* test (SigmaPlot™ , Systat Software Inc, Chicago, IL, US) unless otherwise stated.

## Results

### Chronic liver inflammation is initiated shortly after birth in *Glmp*^*gt/gt*^ mice

**Fig. 1:**
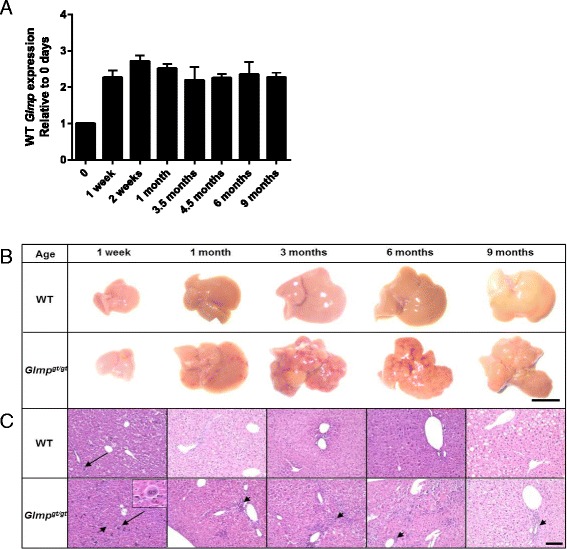
Expression of GLMP in wild-type mice and analysis of the *Glmp* ^*gt/gt*^ phenotype. Wild-type (WT) and *Glmp* ^*gt/gt*^ mice were sacrificed at designated time-points from newborn to 9 months of age, and their livers were extracted. **a** Expression of mRNA for GLMP at different time-points was measured by qPCR. **b** Representative images of WT and *Glmp* ^*gt/gt*^ livers from selected time-points. *Scale bar* 1 cm. **c** Paraffin-embedded liver sections from WT and *Glmp* ^*gt/gt*^ mice from age-matched mice, stained with hematoxylin and eosin, revealed extramedullar hematopoiesis (*arrows*) and infiltration of inflammatory cells (*arrowheads*). *Scale bar* 100 μm

As a background for evaluating the lack of GLMP expression on liver health, we measured the mRNA levels for *Glmp* from birth till 9 months of age. Figure 1a shows a rapid increase in *Glmp* expression within the first week of life, reaching the adult levels by week 2. Next, livers from wild-type (WT) and *Glmp*^*gt/gt*^ mice were analyzed macroscopically and histologically. Figure 1b shows representative liver macro images. No signs of liver injury were observed in newborn mice (data not shown), nor did livers from 1 week old *Glmp*^*gt/gt*^ mice show obvious signs of liver injury. At 1 month of age, however, subcapsular bleeding was observed (Fig. 1b). From the age of 3 months increasing subcapsular contractions appeared, giving a nodular appearance of *Glmp*^*gt/gt*^ livers (Fig. 1b). By the age of 6 months, more distinct nodules were seen; however, at 9 months, the contracted liver surface appeared slightly smoothened compared to 3 and 6 months old *Glmp*^*gt/gt*^ livers (Fig. 1b). Histological examination revealed infiltration of leukocytes as early as 1 week after birth in *Glmp*^*gt/gt*^ livers (Fig. 1c), followed by an increased number of infiltrating inflammatory cells as the animals grew older. A pronounced distortion of the hepatic parenchymal architecture was observed from 1 month of age (Fig. 1c). In addition, the *Glmp*^*gt/gt*^ livers were characterized by wide spread extramedullar hematopoietic cells from the age of 1 week to the age of 6 months (Fig. 1c). Very moderate amounts of such hematological precursors were only seen in 1 week old WT livers as is typical for young individuals [31].

**Fig. 2:**
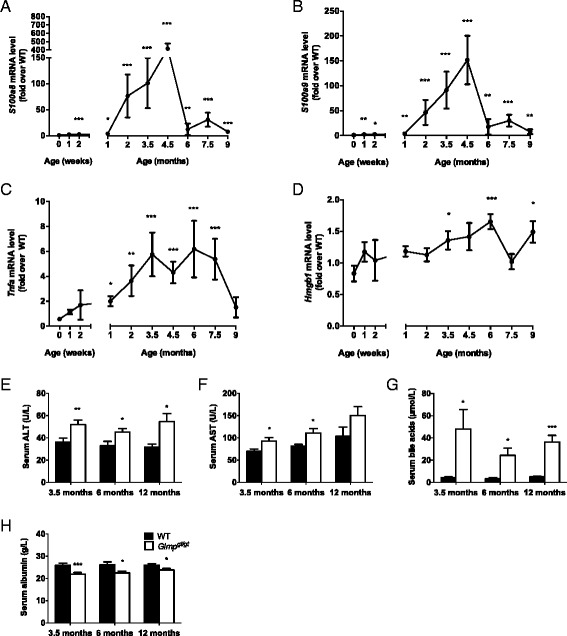
Expression of inflammatory markers and analysis of liver function. Relative mRNA expression of genes involved in inflammation and recruitment of inflammatory cells was analyzed by qPCR and showed age-dependent changes in the expression of **a** *S100a8*, **b** *S100a9*, **c** *Tnfa*, and **d** *Hmgb1* in *Glmp* ^*gt/gt*^ liver (*n* = 4, *p < 0.05, **p < 0.01, ***p < 0.005 vs. WT). Values are presented as mean ± s.e.m. Blood serum was collected from wild-type (WT) and *Glmp* ^*gt/gt*^ mice at 3.5, 6, and 12 months of age. Serum concentrations of **e** alanine transaminase [29], **f** aspartate transaminase (AST), **g** bile acids, and **h** albumin were analyzed (*n* = 5–18, *p < 0.05, **p < 0.01, ***p < 0.005 vs. WT). Values are presented as mean ± s.e.m

Expression of genes involved in inflammation and recruitment of inflammatory cells in newborn *Glmp*^*gt/gt*^ mice was similar to that of WT confirming the observed absence of liver injury in newborn *Glmp*^*gt/gt*^ mice. In support of histological analyses mRNA levels for the inflammation marker S100 calcium-binding protein A8 (*S100a8*) and its interacting partner *S100a9*, both ligands of the Receptor for advanced glycation end-products (RAGE receptor) [32] were markedly elevated in *Glmp*^*gt/gt*^ livers from 1 week of age (Fig. 2a,b). The expression of mRNA for *Tnfa* (Tumor necrosis factor alpha) also increased but did not reach statistical significance until 1 month of age in *Glmp*^*gt/gt*^ livers (Fig. 2c). A substantial increase in the expression levels of these three genes was detected in *Glmp*^*gt/gt*^ livers compared to WT, reaching an apparent peak at 3.5–4.5 months of age followed by reduced expression (Fig. 2a–c). The mRNA expression of a sensor of damage-associated molecular patterns, high-mobility group box 1 (HMGB1), also a ligand of the RAGE receptor [33], showed a slower increase with age peaking at 6 months of age and remaining elevated throughout the observation period (Fig. 2d).

Histological and transmission electron microscopy (TEM) analyses of other organs showed no abnormalities in *Glmp*^*gt/gt*^ mice (Additional file 2: Figure S1).

### Reduced liver function in *Glmp*^*gt/gt*^ mice

The apparent decrease in expression of inflammatory marker genes after 3.5–4.5 months of age in *Glmp*^*gt/gt*^ livers were compared to liver functional analyses. In *Glmp*^*gt/gt*^ mice, modest but significantly elevated levels of serum alanine transaminase [29] (Fig. 2e) and aspartate transaminase (AST) were detected between 3.5 and 12 months of age (Fig. 2f), indicating hepatocyte damage. The relative differences between genotypes were stable with age (Fig. 2e, f). The serum bile acid levels were markedly elevated in *Glmp*^*gt/gt*^ mice compared to WT at all ages studied (Fig. 2g), whereas serum albumin, a marker for hepatic function and the general status of animal health [34], was reduced in *Glmp*^*gt/gt*^ mice compared to WT at all ages studied (Fig. 2h). Serum levels of γ-glutamyl-transferase and serum bilirubin levels remained normal in *Glmp*^*gt/gt*^ mice (data not shown).

### *Glmp*^*gt/gt*^ mice have anemia, thrombocytopenia, and reduced levels of white blood cells

A complete blood analysis was performed on 4.5-month-old WT and *Glmp*^*gt/gt*^ mice. A decrease in concentration of red blood cells (RBC), hemoglobin (HGB), and hematocrit (HCT) and increase in the range of cellular sizes of erythroid cells (RDW) in *Glmp*^*gt/gt*^ mice were observed indicating anemia (Table 1). Furthermore, decreased platelet (PLT) concentration in *Glmp*^*gt/gt*^ mice was detected (Table 1) as well as extended bleeding time (data not shown). The low platelet count was in accordance with the decreased serum levels of TGF-β in *Glmp*^*gt/gt*^ mice, aged 3 weeks–15 months (Additional file 3: Figure S2). In support of the observed increase in infiltrating inflammatory cells in the *Glmp*^*gt/gt*^ liver, a significantly reduction in the levels of circulating white blood cells (WBC) was observed in these mice compared to WT mice (Table 1).

**Table 1:** Blood analyses of 4.5 months old wild-type and *Glmp* ^*gt/gt*^ mice

		Wild-type (*n* = 10)	*Glmp* ^*gt/gt*^ (*n* = 11)
RBC	(×10^12^/L)	9.77 ± 0.08	9.17 ± 0.12***
HGB	(g/dL)	14.01 ± 0.18	13.21 ± 0.18**
HCT	(%)	45.5 ± 0.78	42.67 ± 0.90*
MCHC	(g/dL)	30.85 ± 0.43	30.88 ± 0.37
MCV	(μm^3^)	46.56 ± 0.70	46.68 ± 0.58
RDW	(%)	13.78 ± 0.15	16.30 ± 0.27***
PLT	(×10^10^/L)	84.68 ± 4.63	52.31 ± 6.92**
WBC	(×10^9^/L)	5.08 ± 0.44	2.98 ± 0.29**
Neutrophiles	(×10^9^/L)	0.65 ± 0.06	0.91 ± 0.14
Lymphocytes	(×10^9^/L)	3.86 ± 0.38	1.78 ± 0.18***
Eosinofilic	(×10^9^/L)	0.32 ± 0.05	0.13 ± 0.02**
LUC	(×10^9^/L)	0.14 ± 0.02	0.06 ± 0.02**
Monocytes		N.D.	N.D.
Basofilic		N.D.	N.D.

*p < 0.05; **p < 0.01, ***p < 0.005

*RBC* red blood cells, *HGB* hemoglobin, *HCT* hematocrit, *MCHC* mean corpuscular hemoglobin concentration, *MCV* mean corpuscular volume, *RDW* RBC distribution width, *PLT* platelets, *WBC* white blood cells, *LUC* large unstained cells, *N.D.* not detected

### Liver fibrosis is initiated after birth in *Glmp*^*gt/gt*^ mice

**Fig. 3:**
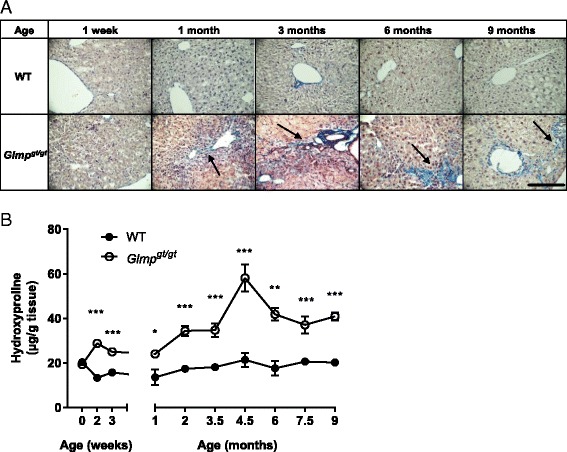
Determination of liver collagen expression and cross-linking. Representative images of paraffin-embedded liver sections from aged-matched wild-type (WT) and *Glmp* ^*gt/gt*^ mice, stained with acid fuchsin orange G (*blue*) (**a**) revealed excess depositions of collagen in *Glmp* ^*gt/gt*^ liver parenchyma from 1 month of age (*arrows*). *Scale bar* 100 μm. **b** Quantification of collagen in age-matched WT and *Glmp* ^*gt/gt*^ livers were determined by analyzing liver hydroxyproline content (*n* = 3–4, *p < 0.05, **p < 0.01, ***p < 0.005 vs. WT). Values are presented as mean ± s.e.m

Staining of liver sections with AFOG showed no increase in collagen staining in *Glmp*^*gt/gt*^ livers compared to WT at 1 week of age (Fig. 3a). However, from 1 month of age, excess collagen was clearly visible in the parenchyma of *Glmp*^*gt/gt*^ livers (Fig. 3a). Liver hydroxyproline levels were quantified as a measure of excess collagen in *Glmp*^*gt/gt*^ livers. Figure 3b shows that in newborn *Glmp*^*gt/gt*^ mice the levels of hydroxyproline did not differ from those of WT mice, demonstrating that *Glmp*^*gt/gt*^ mice were not born with liver fibrosis. Elevated hydroxyproline contents were detected in *Glmp*^*gt/gt*^ livers from 2 weeks, indicating that fibrogenesis had been initiated by this age (Fig. 3b). The highest levels of hydroxyproline were detected in 4.5-month-old *Glmp*^*gt/gt*^ livers, reaching on average a threefold increase compared to WT (Fig. 3b). In accordance with the observed attenuation of inflammation in *Glmp*^*gt/gt*^ livers after 4.5 months (Figs. 1 and 2), the hydroxyproline levels were reduced somewhat after this age (Fig. 3b).

**Fig. 4:**
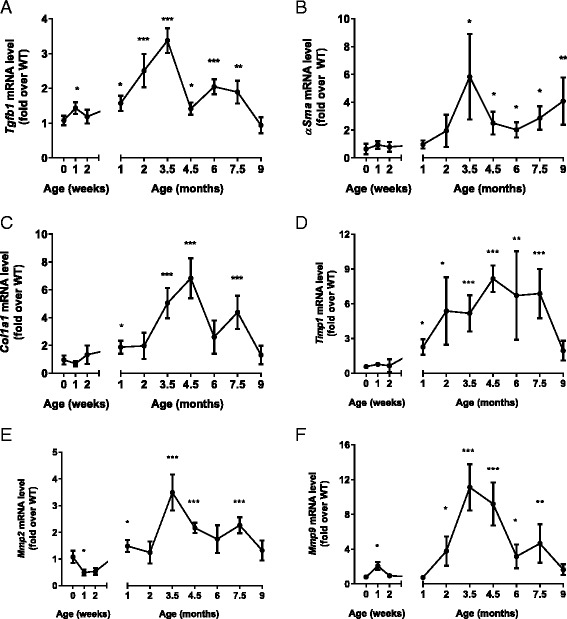
Determination of liver fibrosis markers. Changes in mRNA expressions were assessed by qPCR. Analyses of age-matched mouse livers from wild-type (WT) and *Glmp* ^*gt/gt*^ mice showed altered gene expression of **a** the fibrogenesis inducer *Tgfb1*, **b** the marker for activated hepatic stellate cells *αSma*, **c** fibrillar collagen *Col1a1*, and genes involved in matrix remodeling **d** *Timp1*, **e** *Mmp2*, and **f** *Mmp9* (*n* = 4, *p < 0.05, **p < 0.01, ***p < 0.005 vs. WT). Values are presented as mean ± s.e.m

Gene expression analyses of TGF-β (Fig. 4a), an inducer of fibrogenesis, α-SMA (Fig. 4b), marker of activated hepatic stellate cells, and proteins involved in matrix remodeling collagen 1A1 (COL1A1), matrix metalloproteinase-2 and 9 (MMP2, MMP9) and tissue inhibitor of metalloproteinase-1 (TIMP1) (Fig. 4c–f) revealed an increase in expression peaking at 3.5–4.5 months of age in *Glmp*^*gt/gt*^ livers, where all markers were significantly increased compared to WT. As with the inflammatory markers (Fig. 2a–d) and collagen contents (Fig. 3a, b), the relative differences between the genotypes was reduced after this age (Fig. 4).

### Simultaneous loss and proliferation of hepatocytes in *Glmp*^*gt/gt*^ mice

**Fig. 5:**
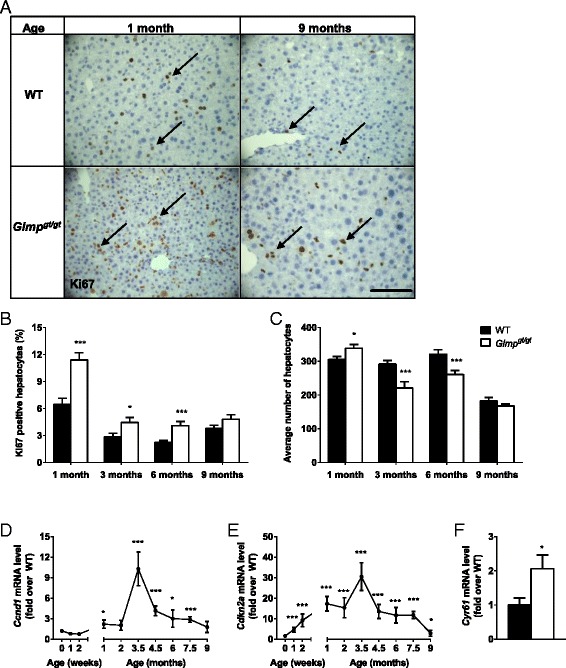
Hepatocyte proliferation and control of the cell cycle. Representative images of liver sections from aged-matched wild-type (WT) and *Glmp* ^*gt/gt*^ mice (**a**) labeled for active Ki67 and counterstained with Mayer’s hematoxylin indicate cell proliferation (*arrows*). *Scale bar* 100 μm. **b** Quantification of proliferating hepatocytes and **c** number of hepatocyte nuclei in aged-matched WT and *Glmp* ^*gt/gt*^ liver sections (*n* = 4, representing six random images and at least 1000 hepatocytes from four individuals/genotype/age). Relative mRNA expression at designated age-points of the proliferation marker **d** *Ccnd1* and one of its inhibitor **e** *Cdkn2a*, and the expression of senescence marker **f** *Cyr61* at 3.5 months were analyzed using qPCR in mouse livers from WT and *Glmp* ^*gt/gt*^ mice (*n* = 4, *p < 0.05, **p < 0.01, ***p < 0.005 vs. WT). Values are presented as mean ± s.e.m

An increased apoptotic activity has been demonstrated in liver from *Glmp*^*gt/gt*^ mice [28]. To explore the regenerative capacity of WT and *Glmp*^*gt/gt*^ livers, paraffin-embedded liver sections were labeled for the proliferation marker Ki67 [35]. Fig. 5a, b shows that the number of proliferating cells is highly increased in 1-month-old *Glmp*^*gt/gt*^ livers compared to WT. A similar tendency, although not as pronounced was observed in 3 and 6 months old mice (Fig. 5b). At 9 months of age, the number of proliferating hepatocytes was comparable between the genotypes (Fig. 5a, b). Semi-quantitative estimation of the total hepatocyte number included in a microscopic field (×40 obj.) showed comparable cell numbers at 1 month of age (Fig. 5c). In *Glmp*^*gt/gt*^ livers from 3- and 6-month-old mice, the number of hepatocytes was lower compared to WT due to enlarged size (Fig. 5c). However, at 9 months of age, *Glmp*^*gt/gt*^ mice appeared to have normalized the number of hepatocytes (Fig. 5c).

To further investigate the proliferative status, the relative expression of Cyclin D1 (CCND1), a key regulator of cell cycle progression [36], was determined. Figure 5d shows an increase in *Ccnd1* expression at 1 month of age in *Glmp*^*gt/gt*^ livers compared to WT, followed by an expression peak at 3.5 months and a reduction in relative gene expression after this age. The expression of Cyclin-dependent kinase inhibitor 2A (*Cdkn2a*), an inhibitor of CCND1, the upregulation of which promotes senescence and growth arrest [37], was significantly upregulated in *Glmp*^*gt/gt*^ livers compared to WT at 1 week of age (Fig. 5e). Similar to the expression of *Ccnd1*, and the other genes involved in inflammation and fibrogenesis, the difference in relative *Cdkn2a* gene expression peaked at 3.5 months followed by a reduction after this age (Fig. 5e). At the peak age of 3.5 months, gene expression analyses of Cysteine-rich angiogenic inducer 61 (*Cyr61*), a marker for senescent cells, showed a significant upregulation in *Glmp*^*gt/gt*^ livers compared to WT (Fig. 5f).

### Oval cell expansion in *Glmp*^*gt/gt*^ livers

**Fig. 6:**
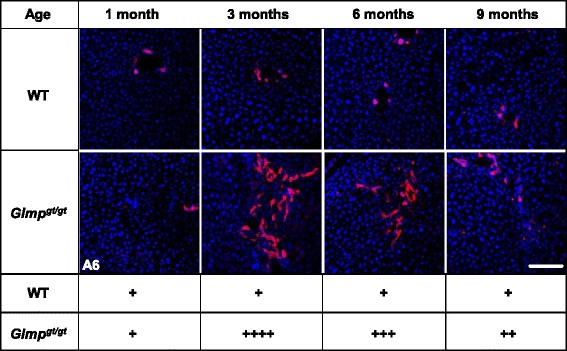
Analysis of oval cell proliferation in *Glmp* ^*gt/gt*^ and wild-type mice. Livers from 1, 3, 6 and 9 months old wild type (WT) and *Glmp* ^*gt/gt*^ mice were fixed and sectioned. Representative immunofluorescence images analyzing expression of the oval cell marker A6 (*red*). Liver sections were counterstained with DAPI (*blue*). Scale bar: 50 μm. Semi-quantitative grading of A6-labeling intensities are indicated by + - signs, ranging from + (control) to ++++ (most intense)

The age-dependent activation of the hepatic oval cell compartment was studied by immunofluorescence staining for the specific marker, A6 [30]. Figure 6 shows similar staining in *Glmp*^*gt/gt*^ and WT livers at 1 month of age. However, at 3 months, a strong increase in A6 staining was detected in *Glmp*^*gt/gt*^ livers, followed by a gradual decrease at 6 and 9 months of age, where the labeling is only slightly increased in *Glmp*^*gt/gt*^ livers compared to WT (Fig. 6).

### Liver tumors develop in old *Glmp*^*gt/gt*^ mice

Systematic evaluation of livers from *Glmp*^*gt/gt*^ mice aged between 12 and 18 months, revealed an increased frequency of liver tumors compared to WT mice (Table 2). Additional file 4: Figure S3 shows representative liver macro images from 18 months old *Glmp*^*gt/gt*^ and WT mice. Hepatocellular tumors (Additional file 4: Figure S3A, B) and hemangioma-like tumors with dilated vessels were detected in the majority of *Glmp*^*gt/gt*^ livers (Additional file 4: Figure S3A,C). In spite of tumor growth, the average liver/body weight ratio was only slightly increased in 18 months old *Glmp*^*gt/gt*^ mice, and no significant differences were detected for spleen/body weight ratio (data not shown). The life expectancy of the *Glmp*^*gt/gt*^ mice appeared to be around 18 months, as the mortality rate of *Glmp*^*gt/gt*^ mice increased between 16 and 18 months of age (data not shown). Liver functional tests, assessed by measuring serum parameters, suggested that liver function in old *Glmp*^*gt/gt*^ mice was comparable to *Glmp*^*gt/gt*^ mice of younger age (Additional file 5: Figure S4 A, B, C, D). Similarly, total liver collagen in *Glmp*^*gt/gt*^ livers did not correlate with increasing incidence of tumor growth (Additional file 6: Figure S5).

**Table 2:** Tumor frequencies in long-lived wild-type and *Glmp* ^*gt/gt*^ mice

Age (months)	Wild-type	*Glmp* ^*gt/gt*^
>12	0/9	4/10
>18	0/10	10/16
>18	0/10	10/16

## Discussion

In this report, the progression of chronic liver injury in *Glmp*^*gt/gt*^ mice across the expected life span is described. In newborn *Glmp*^*gt/gt*^ mice, no signs of liver injury were detected, but chronic liver injury was initiated shortly after birth. At 1 month of age signs of liver damage appeared in the form of subcapsular bleeding, infiltration of inflammatory cells and increased levels of hydroxyproline. Histological analyses detected increased deposition of fibrous collagen in the periportal areas of *Glmp*^*gt/gt*^ liver in accordance with the increased levels of hydroxyproline and increased expression of α-smooth muscle actin (α-SMA) due to activation of stellate cells, the major producers of extracellular matrix components in both healthy and fibrotic liver [38]. The increased presence of inflammatory cells coincided with increased expression levels of mRNA for inflammatory markers and cytokines, matrix modulating enzymes, and their inhibitors. A similar expression pattern for *Ccnd1* and *Cdkn2a* were observed. Further, liver injury was reflected in the elevated serum transaminase levels, mild anemia, increased hepatocyte proliferation, and oval cell activation. Finally, a majority of *Glmp*^*gt/gt*^ mice developed liver cancers after the age of 12 months.

In wild-type mice *Glmp* expression increased rapidly after birth, coinciding with the rapid growth of both liver and the animal. In a recent report, we showed that the liver/body weight ratio more than doubled in the first 2 weeks of life [39]. In the early phase of life, the liver metabolism has to adapt to lactation and in this scenario, the lack of GLMP presumably causes the chronic liver injury leading to the observed fibrosis. In a previous report, we showed increased apoptosis and oxidative stress in *Glmp*^*gt/gt*^ livers leading to hepatocyte loss [28]. Such a loss may be compensated by proliferation of differentiated hepatocytes or by oval cell proliferation and transdifferentiation into hepatocytes. The former typically occurs after mild or limited injuries to the liver by for instance carbon tretrachloride (CCl_4_) [34]. However, in situations of persistent or severe injury leading to insufficient hepatocyte proliferation, oval cells are mobilized to replace lost hepatocytes or to complement an inadequate rate hepatocyte of regeneration [40–43]. Increased hepatocyte proliferation and oval cell mobilization were observed in this study up to the age of 6 months, indicating that lack of GLMP resulted in persistent liver injury. Several rodent models with chronic metabolic liver injuries are associated with proliferation of oval cells in order to compensate for impaired hepatocyte proliferation [42–45]. After 6 months, the oval cell activation declined, and the hepatocyte proliferation appeared to be normalized in *Glmp*^*gt/gt*^ livers compared to WT. The liver injury and fibrosis, however, had not been reversed as shown by the continued increase in the levels of hydroxyproline and HMGB1. The observed continued elevated levels of serum transaminases, increased bile acids and lower serum albumin, often associated with chronic liver disease also supported the notion of continued liver injury [46–48]. Proliferation of liver cells was concomitant with the observed changes in gene expression of the regulator of cell cycle progression, CCND1 and its inhibitor CDKN2A, which increased shortly after birth, reaching a peak around 3.5 months and followed by a decline to normal levels at 9 months. The effect on cell cycle regulation and increased expression of *Cyr61* at 3.5 months indicated cell senescence and initiation of a repair process [49]. The combined effect of these regulators on cell proliferation may be to ensure that activated stellate cells are silenced and damaged cells especially damaged hepatocytes are removed. The increased expression of *Cyr61* indicated that regeneration of the liver is activated and may be the reason for avoiding progression to severe cirrhosis, liver failure, and early death [49]. Using microarray analyses of the *Mdr2*^−/−^ mouse model for chronic biliary injury [40]. Katzenellenbogen et al. showed that DNA replication appeared normal in *Mdr2*^−/−^ liver, while cell division was inhibited, possibly as a protective mechanism from uncontrolled proliferation [41]. At 9 months of age, we detected comparable levels of Ki67 staining and numbers of hepatocytes in *Glmp*^*gt/gt*^ and WT livers, suggesting establishment of a new balance between hepatocyte death and replenishment. Activation of oval cells appeared to be important for maintenance of liver function in *Glmp*^*gt/gt*^ mice. However, oval cells have also been shown to contribute to the development of liver cancer [50, 51], a common end-stage in chronic liver disease [19, 24, 52]. Even though liver functional tests indicated only a mild liver injury and adequate hepatic function in old *Glmp*^*gt/gt*^ mice, the prolonged insult eventually promoted tumorigenesis in about 60 % of 18-month-old mice. A recent study has questioned the true contribution of oval cell expansion in the hepatocyte repopulation after liver injury [53]; however, our data showed that the oval cell compartment was activated at the age where the highest expression of the cell cycle inhibitor CDKN2A was detected [37] and that the total number of hepatocytes normalized after oval cell activation. Further studies will be required to elucidate the role of the oval cells in *Glmp*^*gt/gt*^ liver.

In our first characterization of the *Glmp*^*gt/gt*^ mice, we reported accumulation of lipofuscin and iron in storage vacuoles in *Glmp*^*gt/gt*^ Kupffer cells [28]. Here, we show that the *Glmp*^*gt/gt*^ livers were exposed to chronic inflammation right after birth. Further, we detected mild anemia in *Glmp*^*gt/gt*^ mice, consistent with anemia of inflammation [54, 55]. A study conducted by Nelson et al. [56] showed that iron deposition specifically in Kupffer cells was associated with metabolic liver injury. In a recent study, we have demonstrated dysregulation of glucose and lipid metabolism in *Glmp*^*gt/gt*^ liver and isolated primary hepatocytes [39]. The absence of signs of liver injury in newborn *Glmp*^*gt/gt*^ mice suggests that GLMP may not be essential in mouse prenatal life. At the mRNA level, *Glmp* expression has been shown in mouse embryos [57], suggesting that GLMP might have a role in a biological pathway not active until birth. Dramatic changes in the hepatic gene expression pattern take place right after birth in mice, switching from primarily hematopoietic pathways to metabolic pathways [57]. A disturbed lipid metabolism is a very likely generator of reactive oxygen species (ROS), which are powerful inducers of fibrosis [58]. Lipopolysaccharides (LPSs) leaked from the intestine represent an additional contribution to the development of liver fibrosis by causing inflammation [59–61].

An interesting question is why fibrosis occurs in *Glmp*^*gt/gt*^ liver, and yet it appears to reach a new equilibrium after 6 months, albeit with a high risk of tumor development in old animals. Based on the data presented here showing that hepatocyte proliferation and oval cell proliferation occur simultaneously, we hypothesize that four factors may contribute to the observed fibrosis: the demand for high activity in metabolic pathways after birth, increased influx of LPS from the intestine, the demand for liver growth to accompany the growth of the animal, and the increased need to replace damaged hepatocytes. The high demand for metabolic activity, especially with regard to lipids, which occurs after birth, generates ROS and oxidative stress in *Glmp*^*gt/gt*^ hepatocytes due to their reduced lipid metabolic capacity [39]. This impairs their proliferation potential and leads to apoptosis and influx of inflammatory cells from circulation [17, 19, 20]. Further, in the first 4–5 months the liver has to increase its size to meet the requirements of the growing animal. Simultaneously, increased influx of LPS, secondary to chronic liver injury, is carried via the portal circulation to liver [62–64]. Kupffer cells can be activated by LPS [65] or through ingestion of hepatocyte apoptotic bodies [66], leading to secretion of pro-inflammatory and pro-fibrogenic cytokines like TNF-α [66, 67] and TGF-β [29, 68]. These cytokines are key activators of oval cells and hepatic stellate cells, respectively, in chronically injured liver [17, 20, 69]. In addition, LPS can directly promote stellate cell activation through toll-like receptor 4 (TLR4) [70]. During the growth phase, expansion of the oval cell compartment is required in *Glmp*^*gt/gt*^ liver in order to meet the demand for replenishment of damaged hepatocytes and proliferation for hyperplastic growth. However, around 6 months of age the mice have reached their full body size and so has the liver. When the demand for hyperplastic liver growth is no longer relevant, the *Glmp*^*gt/gt*^ liver appears less dependent on oval cell expansion in maintaining adequate liver function.

## Conclusions

In summary, we have presented the entire liver disease development in *Glmp*^*gt/gt*^ mice, from initiation, progression, and compensation to tumor development over a life span of 18 months. To our knowledge, this is the first in-depth characterization of liver disease development in a transgenic mouse model for a spontaneous, slowly progressing liver fibrosis. We show that liver injury is initiated right after birth in *Glmp*^*gt/gt*^ mice. The injury is mild, but an imbalance between hepatocyte death and proliferation activates the oval cell compartment by the age of 3 months. A new balance between hepatocyte loss and replacement appears to be reached by the age of 9 months; however, the continuous proliferation eventually results in tumorigenesis in *Glmp*^*gt/gt*^ livers. Finally, based on our earlier data, we suggest that metabolic liver injury may be the cause of the liver disease in *Glmp*^*gt/gt*^ mice and propose that this *Glmp*^*gt/gt*^ mouse model may become useful for studies of slowly progressing liver fibrosis and possibly as a model for a yet undescribed lysosomal disorder.

## Additional files

Additional file 1: Table S6. This file provides the sequences of the primer-pairs used for qPCR. (DOC 38 kb) Additional file 2: Figure S1. Extrahepatic tissues show no phenotypic changes. Wild-type (WT) and *Glmp* ^*gt/gt*^ mice were sacrificed 6 months of age, and the lung, kidney, heart, spleen, and colon were extracted, embedded in paraffin and sectioned. (A) Tissue sections were stained with hematoxylin and eosin or (B) acid fuchsin orange G (blue). Scale bars, 200 μm. (C) WT and *Glmp* ^*gt/gt*^ mice were perfusion fixated with 4 % formaldehyde and 2.5 % glutaraldehyde. The lung, kidney, heart, spleen, and colon were extracted, sectioned, and analyzed with transmission electron microscopy. Scale bars, 2 μm. (PDF 403 kb) Additional file 3: Figure S2.
*Glmp* ^*gt/gt*^ mice have reduced serum TGF-β levels. Blood serum was collected from wild-type (WT) and *Glmp* ^*gt/gt*^ mice at 3 weeks, 3 months, and 15 months of age. Serum concentrations of TGF-β were analyzed using enzyme-linked immunosorbent assay (*n* = 5, **p* < 0.05, ***p* < 0.01, ****p* < 0.005 vs. WT). Values are presented as mean ± s.e.m. (PDF 111 kb) Additional file 4: Figure S3. Old *Glmp* ^*gt/gt*^ mice develop liver tumors. Wild-type (WT) and *Glmp* ^*gt/gt*^ mice were sacrificed 18 months of age, and the livers were extracted. (A) Representative images show the presence of a hemangioma-like tumor (arrow) and a hepatocellular tumor (arrowheads) in *Glmp* ^*gt/gt*^ livers. Scale bar, 1 cm. (B) *Glmp* ^*gt/gt*^ livers stained with hematoxylin and eosin revealed tumors of hepatocellular origin (stars mark circumference of tumor) and (C) hemangioma-like tumors with dilated, blood-filled vessels spindle cell proliferation (arrows). Scale bar, 400 μm. (PDF 112 kb) Additional file 5: Figure S4. Serum functional parameters in old mice. Blood serum was collected from wild-type (WT) and *Glmp* ^*gt/gt*^ mice at 18 months of age. Serum concentrations of (A) alanine transaminase [29], (B) aspartate transaminase (AST), (C) bile acids, and (D) albumin were analyzed (*n* = 6–11, **p* < 0.05, ***p* < 0.01, ****p* < 0.005 vs. WT). Values are presented as mean ± s.e.m. (PDF 91 kb) Additional file 6: Figure S5. Total hepatic collagen contents in 18 months old wild-type (WT) and *Glmp* ^*gt/gt*^ mice were assessed by analyzing liver hydroxyproline content (*n* = 4, **p* < 0.05, ***p* < 0.01, ****p* < 0.005 vs. WT). Values are presented as mean ± s.e.m. (PDF 81 kb)

## Abbreviations

α-SMA: alpha- smooth muscle actin
A6: antigen marker for hepatic oval cells
AFOG: acid fuchsin orange G
ALT: alanine transaminase
AST: aspartate transaminase
CCND1: cyclin D1
CDKN2A: cyclin-dependent kinase inhibitor 2A
COL1A1: collagen 1a1
CYR61: cysteine-rich angiogenic inducer 61
GLMP: glycosylated lysosomal membrane protein
HCT: hematocrit
HGB: hemoglobin
HMGB1: high-mobility group box 1 protein
Ki67: antigen Ki67
LPS: lipopolysaccharide
MDR2^−/−^: knockout of the *mdr2* P-glycoprotein gene
MMP2: matrix metallopeptidase 2
MMP9: matrix metallopeptidase 9
PLT: blood platelet
RAGE receptor: receptor for advanced glycation end-products
RBC: red blood cells
RDW: red cell distribution width
S100a8: S100 calcium-binding protein A8
S100a9: S100 calcium-binding protein A9
TEM: transmission electron microscopy
TGF-β: tumor growth factor beta
TIMP1: TIMP metallopeptidase inhibitor 1
TLR4: toll-like receptor 4
TNFα: tumor necrosis factor alpha
WBC: white blood cell
