# Supplementary material for: Age-dependent development of liver fibrosis in Glmpgt/gt mice
Source: Fibrogenesis Tissue Repair. 2016 Apr 28;9:5. doi: 10.1186/s13069-016-0042-4 (PMC4852418; doi:10.1186/s13069-016-0042-4)
Supplement: Additional file 1: Table S6. — This file provides the sequences of the primer-pairs used for qPCR. (DOC 38 kb) [file 13069_2016_42_MOESM1_ESM.doc]

| Target gene | Description | 5'-Primer | 3'-Primer |
| --- | --- | --- | --- |
| *α-Sma* | Alpha-smooth muscle actin | CCACCGCAAATGCTTCTAAGT | GGCAGGAATGATTTGGAAAGG |
| *β-actin* | Beta-actin | AGCCATGTACGTAGCCATCC | GCTGTGGTGGTGAAGCTGTA |
| *Ccnd1* | Cyclin D1 | TTTCTTGTAGCGGCCTGTTGT | CGGAACACTAGAACCTAACAGATTAAATG |
| *Cdkn2a* | Cyclin-dependent kinase inhibitor 2A | CCCACTCCAAGAGAGGGTTT | TCTGCACCGTAGTTGAGCAG |
| *Col1a1* | Collagen, type I, alpha 1 | GCTTCACCTACAGCACCCTTGTGG | GAGGGAGTTTACACGAAGCAGGCAG |
| *Cyr61* | Cysteine-rich, Angiogenic inducer, 61 | AGTGCCGCCTGGTGAAAGAGA | CACGCAGGAGCCGCAGTATTT |
| *eEF2* | Eukaryotic translation elongation factor 2 | CCATCGCTGAACGCATCAAG | CAGGCCAGAACCAAAGCCTA |
| *Glmp* | Glycosylated lysosomal membrane protein | GCTTCTCCTCGTGGAAACCAT | CGGCAGGTGCATATTCATCA |
| *Hmgb1* | High mobility group box 1 | GGCTGACAAGGCTCGTTATG | GGGCGGTACTCAGAACAGAA |
| *Mmp2* | Matrix metallopeptidase 2 | TGATAACCTGGATGCCGTCGT | TGCTTCCAAACTTCACGCTCT |
| *Mmp9* | Matrix metallopeptidase 9 | CTTTGAGTCCGGCAGACAAT | TTCCAGTACCAACCGTCCTT |
| *S100a8* | S100 calcium binding protein A8 | CCGTCTTCAAGACATCGTTTGA | GTAGAGGGCATGGTGATTTCCT |
| *S100a9* | S100 calcium binding protein A9 | ATACTCTAGGAAGGAAGGACACC | TCCATGATGTCATTTATGAGGGC |
| *Tgfb1* | Transforming growth factor β1 | GCACCATCCATGACATGAACC | AAGTCAATGTACAGCTGCCGC |
| *Tnfa* | Tumor necrosis factor alpha | CAAACCCTGGTATGAGCCCAT | ACCCATTCCCTTCACAGAGCA |
| *Timp1* | Tissue inhibitor of metalloproteinases 1 | GGCATCCTCTTGTTGCTATCACT | CTTATGACCAGGTCCGAGTTGC |
|  |  |  |  |

**Additional file 1.** Primers used for qPCR.
