# Supplementary figures and images for: Age-dependent development of liver fibrosis in Glmpgt/gt mice
Source: Fibrogenesis Tissue Repair. 2016 Apr 28;9:5. doi: 10.1186/s13069-016-0042-4 (PMC4852418; doi:10.1186/s13069-016-0042-4)

Additional file 2: Figure S1

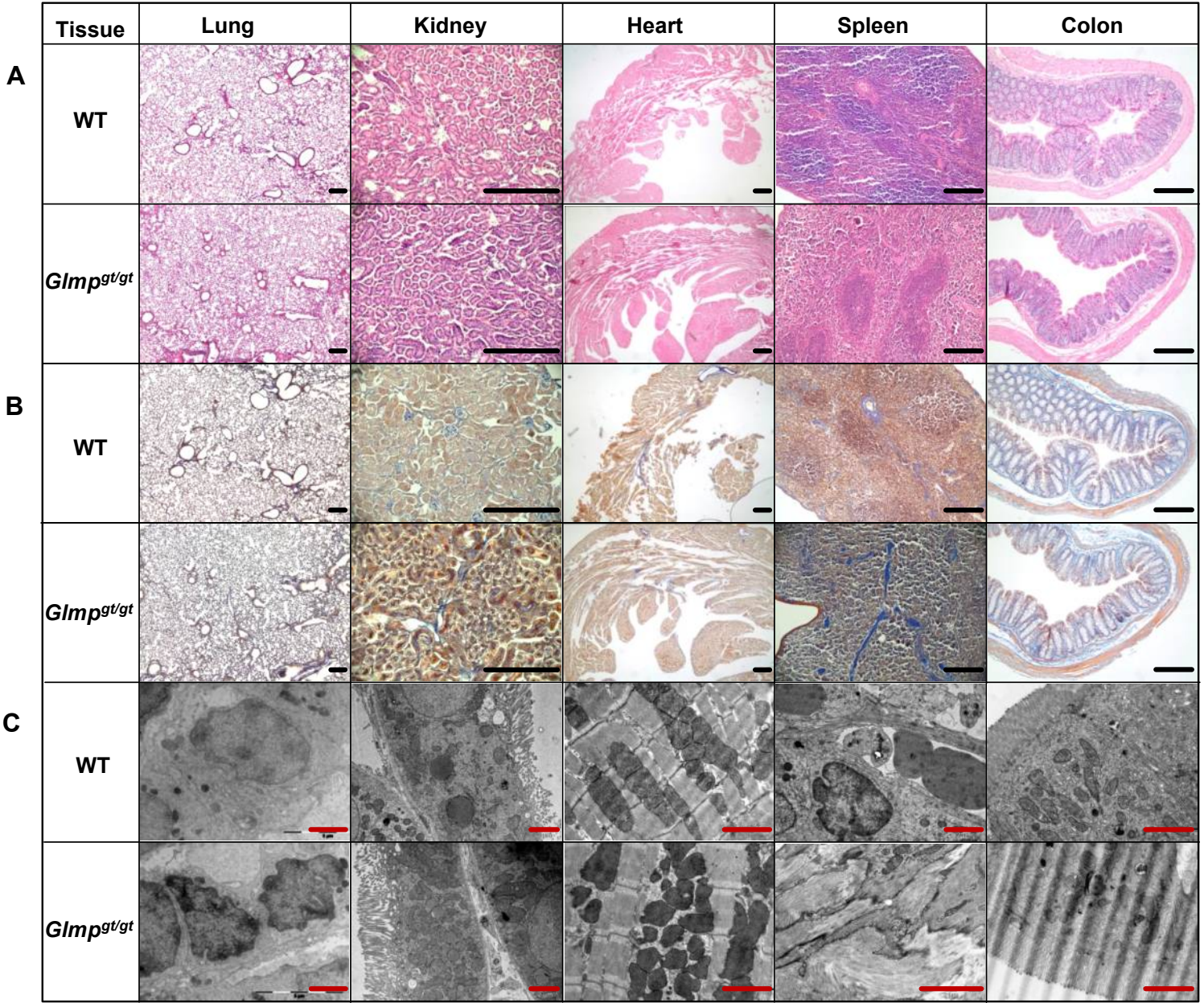

Supplement: Additional file 2: Figure S1. — Extrahepatic tissues show no phenotypic changes. Wild-type (WT) and Glmp gt/gt mice were sacrificed 6 months of age, and the lung, kidney, heart, spleen, and colon were extracted, embedded in paraffin and sectioned. (A) Tissue sections were stained with hematoxylin and eosin or (B) acid fuchsin orange G (blue). Scale bars, 200 μm. (C) WT and Glmp gt/gt mice were perfusion fixated with 4 % formaldehyde and 2.5 % glutaraldehyde. The lung, kidney, heart, spleen, and colon were extracted, sectioned, and analyzed with transmission electron microscopy. Scale bars, 2 μm. (PDF 403 kb) [file 13069_2016_42_MOESM2_ESM.pdf]

### Additional file 3: Figure S2

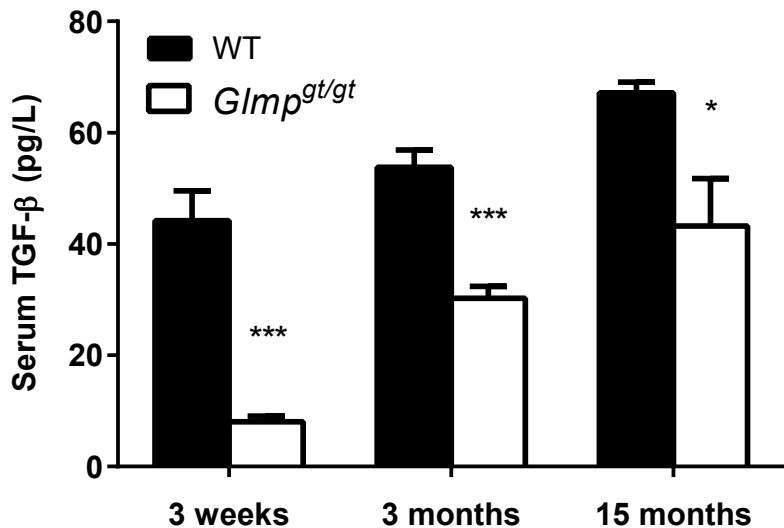

Supplement: Additional file 3: Figure S2. — Glmp gt/gt mice have reduced serum TGF-β levels. Blood serum was collected from wild-type (WT) and Glmp gt/gt mice at 3 weeks, 3 months, and 15 months of age. Serum concentrations of TGF-β were analyzed using enzyme-linked immunosorbent assay (n = 5, *p < 0.05, **p < 0.01, ***p < 0.005 vs. WT). Values are presented as mean ± s.e.m. (PDF 111 kb) [file 13069_2016_42_MOESM3_ESM.pdf]

## Additional file 4: Figure S3

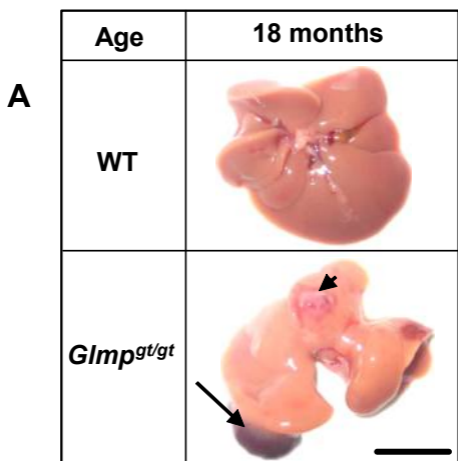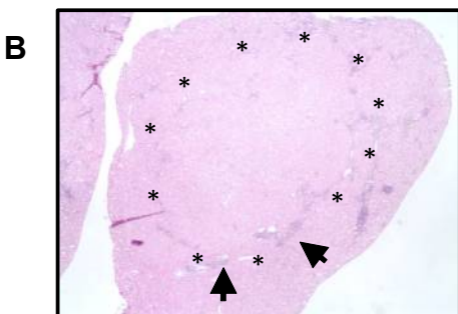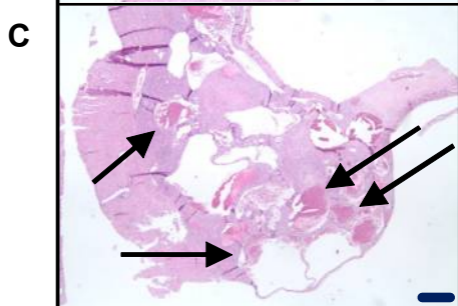

Supplement: Additional file 4: Figure S3. — Old Glmp gt/gt mice develop liver tumors. Wild-type (WT) and Glmp gt/gt mice were sacrificed 18 months of age, and the livers were extracted. (A) Representative images show the presence of a hemangioma-like tumor (arrow) and a hepatocellular tumor (arrowheads) in Glmp gt/gt livers. Scale bar, 1 cm. (B) Glmp gt/gt livers stained with hematoxylin and eosin revealed tumors of hepatocellular origin (stars mark circumference of tumor) and (C) hemangioma-like tumors with dilated, blood-filled vessels spindle cell proliferation (arrows). Scale bar, 400 μm. (PDF 112 kb) [file 13069_2016_42_MOESM4_ESM.pdf]

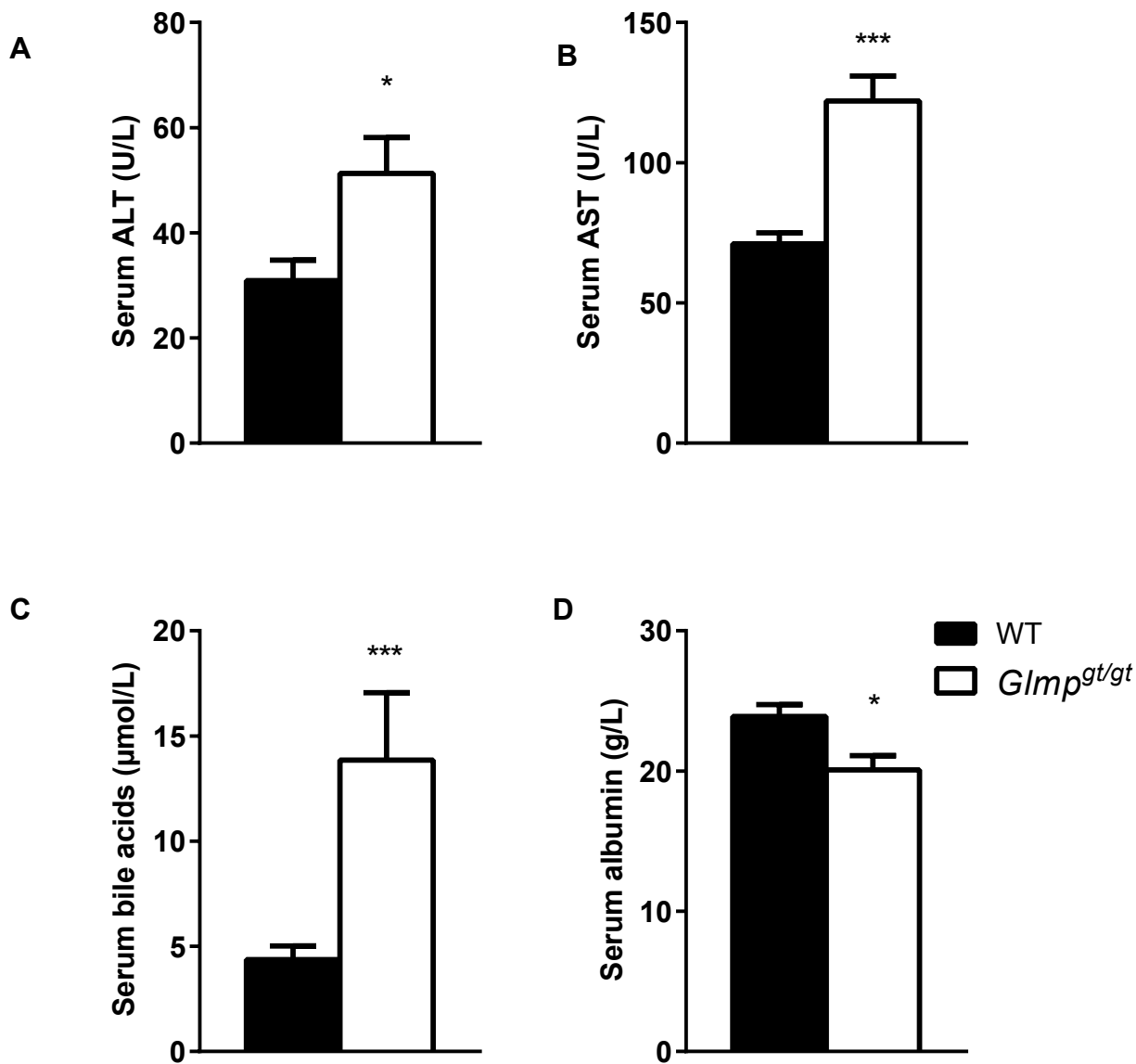

Supplement: Additional file 5: Figure S4. — Serum functional parameters in old mice. Blood serum was collected from wild-type (WT) and Glmp gt/gt mice at 18 months of age. Serum concentrations of (A) alanine transaminase [29], (B) aspartate transaminase (AST), (C) bile acids, and (D) albumin were analyzed (n = 6–11, *p < 0.05, **p < 0.01, ***p < 0.005 vs. WT). Values are presented as mean ± s.e.m. (PDF 91 kb) [file 13069_2016_42_MOESM5_ESM.pdf]

## Additional file 6: Figure S5

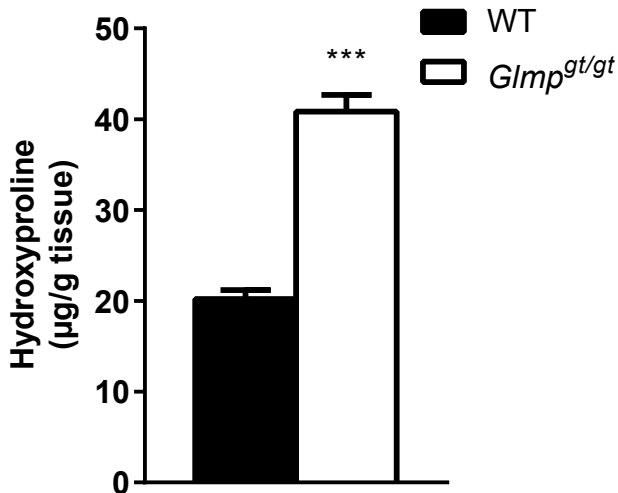

Supplement: Additional file 6: Figure S5. — Total hepatic collagen contents in 18 months old wild-type (WT) and Glmp gt/gt mice were assessed by analyzing liver hydroxyproline content (n = 4, *p < 0.05, **p < 0.01, ***p < 0.005 vs. WT). Values are presented as mean ± s.e.m. (PDF 81 kb) [file 13069_2016_42_MOESM6_ESM.pdf]
